# Supplementary material for: DSResSol: A Sequence-Based Solubility Predictor Created with Dilated Squeeze Excitation Residual Networks
Source: Int J Mol Sci. 2021 Dec 17;22(24):13555. doi: 10.3390/ijms222413555 (PMC8704505; doi:10.3390/ijms222413555)
Supplement: Supplementary file 1 [file ijms-22-13555-s001.zip › ijms-1463816-supplementary.pdf]

Supplementary Information

DSResSol: A sequence-based protein solubility predictor created with dilated squeeze excitation residual networks

Mohammad Madani[1], Kaixiang Lin[2], Anna Tarakanova[1,3*]

[1]Department of Mechanical Engineering, University of Connecticut, Storrs, CT, United States
[2]Department of Computer Science & Engineering, University of Connecticut, Storrs, CT, United States
[3]Department of Biomedical Engineering, University of Connecticut, Storrs, CT, United States
***Corresponding author:**
anna.tarakanova@uconn.edu

Performance of 10 models obtained from 10-fold cross validation for DSResSol (1) and DSResSol (2)

The validation accuracy for each model is obtained from validation data. We utilize 10-fold cross validation for the training process. The training data is divided into 10 parts: 9 parts for training and 1 part for validation, alternatively in each cross-validation. Thus, in each cross-validation training, we use 10% of the training data as validation data and the remaining 90% of the data for the training process.

SI Table 1. Performance comparison for 10 models obtained from 10-fold cross-validation for DSResSol (1) models on both independent test sets. Red blocks are for the first test set and blue blocks are for the second test set. Validation accuracy is on validation data.

Model	Training ACC	Valid ACC	ACC	Precision	Recall	F-1 score	ACC	Precision	Recall	F-1 score
1	0.761	0.753	0.745	0.748	0.745	0.745	0.549	0.553	0.542	0.545
2	0.764	0.762	0.749	0.753	0.748	0.749	0.551	0.550	0.547	0.548
3	0.763	0.758	0.741	0.748	0.741	0.746	0.539	0.538	0.549	0.544
4	0.765	0.761	0.751	0.754	0.750	0.751	0.556	0.558	0.551	0.553
5	0.769	0.759	0.744	0.748	0.743	0.744	0.536	0.537	0.542	0.539
6	0.765	0.760	0.742	0.747	0.743	0.744	0.541	0.539	0.550	0.541
7	0.759	0.758	0.750	0.754	0.749	0.751	0.549	0.551	0.549	0.548
8	0.774	0.768	0.745	0.749	0.743	0.746	0.552	0.550	0.547	0.537
9	0.771	0.769	0.749	0.753	0.747	0.750	0.557	0.558	0.542	0.552
10	0.768	0.758	0.743	0.748	0.743	0.744	0.532	0.529	0.541	0.529

SI Table 2. Performance comparison for 10 models obtained from 10-fold cross-validation for DSResSol (2) models on both independent test sets. Red blocks are for the first test set and blue blocks are for the second test set. Validation accuracy is on validation data.

Model	Training ACC	Valid ACC	ACC	Precision	Recall	F-1 score	ACC	Precision	Recall	F-1 score
1	0.814	0.805	0.789	0.783	0.790	0.787	0.619	0.608	0.611	0.610
2	0.816	0.799	0.787	0.786	0.789	0.787	0.620	0.612	0.613	0.612
3	0.814	0.802	0.791	0.788	0.789	0.788	0.621	0.618	0.601	0.611
4	0.809	0.808	0.796	0.788	0.790	0.789	0.629	0.620	0.616	0.618
5	0.808	0.810	0.787	0.784	0.788	0.786	0.608	0.610	0.599	0.605
6	0.810	0.799	0.793	0.789	0.787	0.788	0.625	0.622	0.616	0.619
7	0.806	0.790	0.791	0.787	0.790	0.789	0.629	0.622	0.618	0.621
8	0.819	0.806	0.790	0.784	0.787	0.786	0.592	0.598	0.607	0.601
9	0.820	0.795	0.784	0.786	0.788	0.787	0.609	0.608	0.603	0.604
10	0.818	0.811	0.785	0.787	0.785	0.786	0.617	0.614	0.612	0.613
